# Challenges in the Caesarean Section of a Severely Kyphotic Parturient

**DOI:** 10.1155/2016/8405052

**Published:** 2016-03-15

**Authors:** Manisha Chhetry, Basudeb Banerjee, Shanti Subedi, Narayan Bahadur Gharti Chhetri, Yogendra Gupta

**Affiliations:** ^1^Department of Obstetrics and Gynecology, Nobel Medical College and Teaching Hospital, Kanchanbari, Biratnagar 5, Morang Eastern Region of Nepal, Biratnagar 56700, Nepal; ^2^Department of Orthopedics and Trauma, Nobel Medical College and Teaching Hospital, Kanchanbari, Biratnagar 5, Morang Eastern Region of Nepal, Biratnagar 56700, Nepal

## Abstract

Caesarean section in a severely kyphotic patient presents with unique challenges. We report a case of obstructed labor in case of a pregnant lady with severe kyphosis of spine that was managed by caesarean section. Lateral recumbent position with adequate assistance and paramedian or vertical skin incision was used and found to provide good exposure. Baby was delivered by lower segment uterine incision by reverse breech extraction. Postpartum hemorrhage was managed with uterotonics and bilateral uterine artery ligation. Tubal ligation though advised was refused by the patient. Prolonged catheterization was done in view of obstructed labor. Postoperative period was uneventful.

## 1. Introduction

Any change in the normal spinal curvature has significant effects on cardiorespiratory physiology and may cause considerable changes in pelvic anatomy which may alter the course of labor and delivery. Cardiorespiratory complications due to kyphoscoliosis and hyperkyphosis of thoracic spine are well studied [1–6]. Primary pathology in lumbar spine leading to kyphosis in this region has overall effect on spinal balance and pelvic obliquity.

Pregnancy in such deformed spine imposes special challenges in management. There is altered growth of gravid uterus and unpredictable delivery [7]. Patients may have uncomplicated vaginal deliveries or may also need operative intervention in the form of instrumental deliveries or caesarean section [8, 9]. In extreme cases of neglected labor, patients may present with obstructed labor requiring caesarean section. Under such circumstances, special attention should be paid to all aspects of surgery starting from anesthesia, patient positioning, skin and uterine incision to delivery of baby. and postoperative care and followup [7].

We report a case of obstructed labor in case of a pregnant lady with severely deformed spine that was managed by caesarean section and have discussed the challenges that we faced while managing the case.

## 2. Case Presentation

We report a case of 24-year-old primigravida at 39-week period of gestation with severe kyphosis who was referred to our centre in second stage of labor with features of obstructed labor.

This patient was resident of remote hilly district who had history of physical assault at age of five years of life leading to vertebral fractures. She was managed with one month of bed rest and then she was mobilized without any support. She regained functional mobility but developed a hunchback.

**Figure 1:**
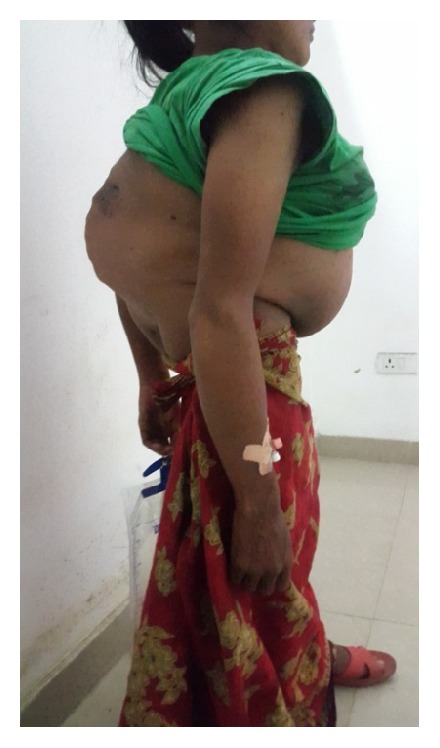
Clinical photograph showing large gibbus over lower back.

**Figure 2:**
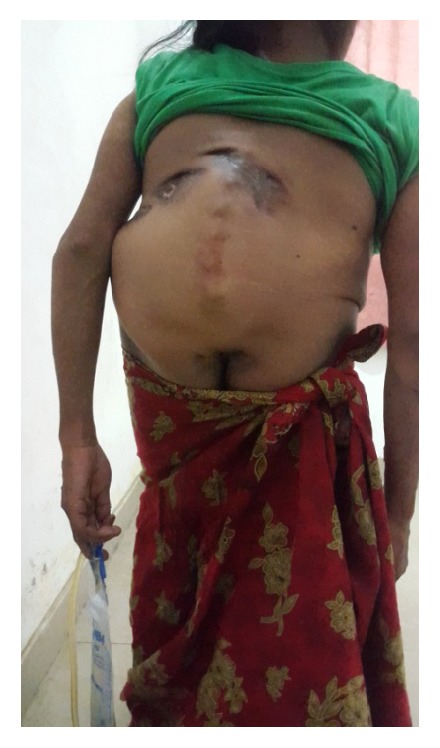
Clinical photograph showing multiple healed sinuses over back.

**Figure 3:**
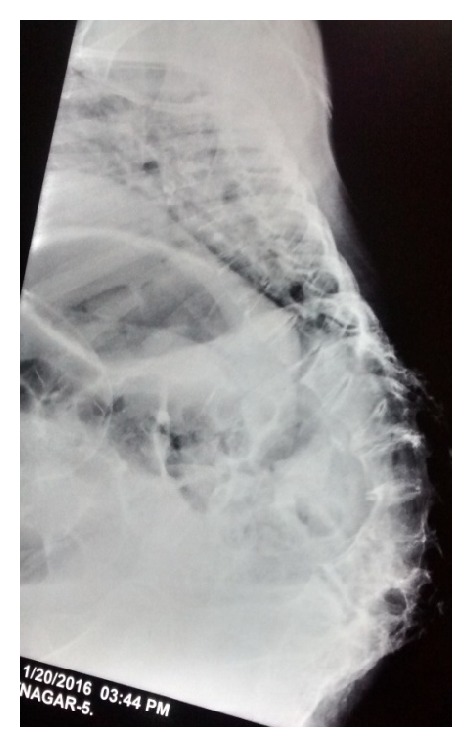
X-ray of spine showing kyphosis in lumbar region and lordosis in thoracic region.

At ten years of age, she developed fever, hemoptysis, swelling over back with pus like discharge, and weight loss for which she was admitted in another centre for one month and was diagnosed as a case of Pott's spine and treated with antitubercular drugs. She did not receive any formal education and used to do all household chores with no restriction in daily activities. She was married at the age of 20 years and conceived spontaneously. She became aware of pregnancy after she felt quickening at four months of gestation and went to the nearest primary health centre which was at a distance of two hours from her home on foot. She was advised to visit a better centre for risk assessment and safe confinement of pregnancy but due to monetary problem she decided to go only after term. She presented to the primary health centre at 39 weeks after one day of labor pain and leaking per vaginum for 48 hours, at around 12 pm midday where she was found to be fully dilated and was immediately referred. She arrived at our facility at 12 midnight and when examined, she was of short stature with height of 4 feet and thin built weighing 33 kgs. Her back was severely kyphotic with large gibbus in the lumbosacral region and multiple healed sinus in the back (Figures 1 and 2). She was exhausted due to prolonged labor and her blood pressure was 150/100 mm of hg. The anterior abdominal wall was markedly protuberant and the axis of the term size uterus showed corresponding anterior angulation. The presentation of the fetus was cephalic with right occiput transverse position and the cardiotocograph was reassuring. Her per vaginum findings were consistent with those of kyphotic pelvis with features of obstructed labor with flat sacrum, narrow outlet, and subpubic angle, the station of the head was −2, there was a large caput succedaneum and moulding, and the liquor was meconium stained. The patient was dyspneic and occasional crepitations could be auscultated in bilateral lung fields and normal cardiac auscultation. Since the patient presented in advanced labor pulmonary function, tests could not be done prior to delivery. As vaginal delivery was not possible, she was immediately taken up for emergency caesarean section. Since the deformity was present in the lumbosacral region with no previous evaluation, spinal anesthesia was not contemplated. General anesthesia was given by using rapid sequence induction with propofol. Caesarean section was done by giving paramedian skin incision. Lower segment uterine incision was used and baby was delivered by reverse breech extraction. It was a 3.7 kg male baby, vigorous at birth, but was admitted in nursery for observation for meconium aspiration syndrome. There was atonic postpartum hemorrhage which was managed with uterine massage, uterotonics, and bilateral uterine artery ligation. Intraoperatively, one pint of blood was transfused. Keeping in view the risk to the health of the patient in subsequent pregnancy, even option of tubal ligation was discussed with the patient and her husband prior to caesarean but they refused to give consent for ligation. Intraoperatively, bladder was found to be edematous and pulled up so catheterization was done for 14 days. Postoperative period was uneventful. Blood pressure normalized after delivery and patient was able to ambulate from second postoperative day. Postoperative hemoglobin was 9.4 mg/dL. Baby was shifted out from nursery on second day and breastfeeding was initiated. Suture was removed on tenth day and wound healing was good. X-ray done after delivery showed reversal of normal curvature of spine with lordosis in thoracic region and kyphosis in lumbar region (Figure 3). Pulmonary function test showed mild restrictive pattern. Patient was discharged on persistent request on 10th postoperative day with catheter in situ and she was advised to follow up at the primary health centre as she cited inability to commute to our centre.

## 3. Discussion

Pregnancy in a severely kyphotic patient is rarely encountered nowadays. The incidence quoted in the early literature varies from 1 in 1470 to 1 in 12,000 [10] though lately incidence of 0.072% has been reported in a study done by Chopra et al. [9]. Etiology is varied with common causes being tuberculosis, trauma, neuromuscular disease like poliomyelitis, and connective tissue disorders [9, 11] to name a few. The striking feature in this case was that in spite of severe deformity there was no cardiorespiratory embarrassment during pregnancy unlike previous reports [3, 6], although Chau and Lee [8] also reported no cardiorespiratory compromise in their case series.

The presence of kyphosis in the lumbar region has direct effect on lower abdomen and pelvis. If the deformity of the spine is significant, there is approximation of the ribcage to iliac crest resulting in the reduction of the available room in the abdomen [7]. As a result, there is an acute angulation of the growing uterus resulting in a pendulous abdomen. Due to distorted anatomy, caesarean section in such a severely kyphotic patient presents with unique challenges.

The first problem encountered in this case was deciding on the positioning of the patient. Patient could not be placed in supine position due to the obvious deformity, so the entire surgery was performed in right lateral recumbent position with three pillows beneath the patient and two assistants centralizing the gravid uterus. In previous case reports, most of them do not mention the patient positioning; only in one report by Berge [7] it has been mentioned that anesthesia was induced with patient in semirecumbent position.

Similarly after anticipating difficult surgery, vertical paramedian skin incision was given. This approach was logical because in any difficult surgery the vertical skin incision provides wider view and easy accessibility to viscera and can be easily extended if need arises. Other case reports [7] have described similar approach.

Due to the pathology discussed previously, there was acute forward angulation of the uterus making the approach to lower segment difficult, but somehow uterine incision could be given in the lower segment. Had the approach to lower segment failed, we had also planned on a classical incision. In a case series reported by Chau and Lee [8], out of the total twenty-five caesarean sections carried out in kyphotic patients, classical incision was given in twelve cases as lower segment was inaccessible. Incision on the fundus and even on the posterior surface of the uterus has been described [8] for delivery of the fetus. In another case series reported by Kopenhager [10], only one out of twenty-five kyphotic patients undergoing caesarean needed a classical incision while, out of 22, no patients needed classical incisions in study done by Chopra et al. [9]. Berge [7] also reported a case where classical incision was given due to inaccessibility of lower segment. The authors are of the view that in case of any similar difficult caesarean the baby should be delivered by incision on the most easily accessible part of the uterus.

The lower segment was thick and edematous as the patient had been in the second stage of labor for more than twelve hours, most of the liquor had drained, and the scanty liquor present was meconium stained, and head was deeply impacted making delivery by vertex difficult. Henceforth baby was delivered using Patwardhan's technique or the reverse breech extraction. Studies have reported the success of this technique in delivering deeply impacted head in obstructed labor [12, 13].

There was atonic postpartum hemorrhage which was managed with uterine massage, uterotonics, and bilateral uterine artery ligation. The atonicity could be due to the prolonged labor especially the second stage and prolonged leaking and also due to possible uterine inertia which has been reported in cases of kyphotic patients most likely due to the acute angulation of uterus which causes the fetal axis to divert from the axis of the birth canal [7, 8].

Consideration for permanent sterilization by tubal ligation should also be made for this group of patients because pregnancy poses a significant increase in morbidity and mortality with possible worsening of disease. Furthermore if delivery was done using a classical incision, risk of rupture in subsequent pregnancy is high.

Caesarean section in a severely kyphotic patient presents with unique challenges. Lateral recumbent position with adequate assistance and paramedian or vertical skin incision should preferably be used. Baby should be delivered by incision on the most easily accessible part of the uterus. PPH should be anticipated and the obstetrician should be prepared for the same. We believe permanent sterilization by tubal ligation should be discussed with the couple prior to the operation. In the event of future pregnancy, the patient should be advised of the need for strict supervision at a tertiary care centre with a multidisciplinary approach and the need for preplanned elective caesarean with proper preoperative anesthetic assessment.

## References

[1] Bergofsky E. H., Turino G. M., Fishman A. P. (1959). Cardiorespiratory failure in kyphoscoliosis. *Medicine*.

[2] Gamzu R., Shenhav M., Fainaru O., Almog B., Kupferminc M., Lessing J. B. (2002). Impact of pregnancy on respiratory capacity in women with muscular dystrophy and kyphoscoliosis. A case report. *The Journal of Reproductive Medicine*.

[3] Hanley T., Platts M. M., Clifton M., Morris T. L. (1958). Heart failure of the hunchback. *The Quarterly Journal of Medicine*.

[4] Mitic-Milikic M. (1996). Pulmonary function in persons with kyphoscoliosis. *Srpski Arhiv za Celokupno Lekarstvo*.

[5] Harrison R. A., Siminoski K., Vethanayagam D., Majumdar S. R. (2007). Osteoporosis-related kyphosis and impairments in pulmonary function: a systematic review. *Journal of Bone and Mineral Research*.

[6] Fearl C. L. (1950). Kyphoscoliosis and pregnancy; its cardiorespiratory implications. *Transactions of the Pacific Coast Obstetrical and Gynecological Society*.

[7] Berge J. E. (1962). Pregnancy associated with severe kyphoscoliosis of the thoracic spine. *The Journal of Obstetrics and Gynaecology of the British Empire*.

[8] Chau W., Lee K. H. (1970). Kyphosis complicating pregnancy. *Journal of Obstetrics and Gynaecology of the British Commonwealth*.

[9] Chopra S., Adhikari K., Agarwal N., Suri V., Sikka P. (2011). Kyphoscoliosis complicating pregnancy: maternal and neonatal outcome. *Archives of Gynecology and Obstetrics*.

[10] Kopenhager T. (1977). A review of 50 pregnant patients with kyphoscoliosis. *British Journal of Obstetrics and Gynaecology*.

[11] Rocke D. A., Moodley J., Datta S. (1996). Orthopedic problems: kyphoscoliosis. *Anesthetic and Obstetric Management of High Risk Pregnancy*.

[12] Levy R., Chernomoretz T., Appelman Z., Levin D., Or Y., Hagay Z. J. (2005). Head pushing versus reverse breech extraction in cases of impacted fetal head during cesarean section. *European Journal of Obstetrics Gynecology and Reproductive Biology*.

[13] Khosla A. H., Dahiya K., Sangwan K. (2003). Cesarean section in a wedged head. *Indian Journal of Medical Sciences*.

